# Outcomes of the LEAP feasibility trial—A low-threshold, exercise programme with protein supplementation to target frailty and poor physical functioning in people experiencing homelessness and addiction issues

**DOI:** 10.1371/journal.pone.0301926

**Published:** 2024-05-31

**Authors:** Fiona Kennedy, Clíona Ní Cheallaigh, Roman Romero-Ortuno, Suzanne L. Doyle, Julie Broderick

**Affiliations:** 1 Discipline of Physiotherapy, School of Medicine, Trinity College Dublin, Dublin, Ireland; 2 St James’s Hospital, Dublin, Ireland; 3 School of Medicine, Trinity College Dublin, Dublin, Ireland; 4 Discipline of Medical Gerontology, School of Medicine, Trinity College Dublin, Dublin, Ireland; 5 Mercer’s Institute for Successful Ageing, St James’s Hospital, Dublin, Ireland; 6 School of Biological, Health and Sports Sciences, Technological University Dublin, Dublin, Ireland; Università degli Studi di Milano: Universita degli Studi di Milano, ITALY

## Abstract

**Background:**

People experiencing homelessness are more likely to experience poor health with physical functioning deficits and frailty commonly reported. It is not well known how strategies to target physical functioning deficits and frailty work in practice in this group. The primary aim of this study was to explore the feasibility of an exercise intervention with protein supplementation to target physical functioning and frailty in people experiencing homelessness evaluated by recruitment and retention rates, adherence to the exercise sessions and protein supplement, adverse effects, programme feedback and characteristics of non-returners, sporadic and frequent attenders. The secondary aim was to evaluate changes in effectiveness outcomes of grip strength, muscle mass, lower extremity physical function, pain, frailty, and risk of malnutrition.

**Method:**

This prospective single-arm study evaluated the feasibility of a 16-week rolling, low-threshold, ‘drop-in’ once weekly exercise programme with protein supplementation. The main recruitment site was a day-service centre for people who are homeless. Feasibility was assessed by the recruitment and retention rates, adherence to the exercise sessions and protein supplement as well as adverse effects, programme feedback and evaluation of characteristics of non-returners, sporadic (≤50% of available sessions) and frequent attenders (≥50% of available sessions). Effectiveness outcomes included pain (Visual Analogue Scale), physical functioning and performance (hand-grip dynamometry, limb circumference, the Short Physical Performance Battery), frailty (SHARE-FI and Clinical Frailty Scale) and nutritional status (Mini Nutritional Assessment).

**Results:**

Thirty-one participants were recruited mean (SD) age 45(16) years. There was a recruitment rate of a median (IQR) of 2(1–3) new participants per week. The retention rate was 45% (n = 14) to the main recruitment site. Adherence to the exercise sessions and nutritional intervention was 90% and 100% respectively. Three adverse events were recorded during 74 interventions over the 16-week programme. The acceptability of the programme was highlighted in participant feedback. Characteristics of frequent returners (≥50%) were older age, female, more stably housed and more stable in addiction. The programme did not induce any changes in effectiveness outcomes.

**Conclusion:**

The feasibility of this programme was demonstrated. Overall, the programme was well received with higher retention rates in older participants, females, those more stably housed and those stable in addiction. A higher powered, more intense programme is needed to demonstrate programme effectiveness.

## Introduction

Inclusion health is an approach, which aims to prevent and address health and social inequalities of vulnerable people such as people experiencing homelessness [1, 2]. The collision of disease risk factors with poverty, constant stressors and social exclusion results in a markedly elevated rate of non-communicable diseases in this population [3] and a mortality rate that is almost eight times higher than the average for men, and nearly 12 times higher for women [4].

Accelerated ageing and earlier geriatric conditions such as falls, poor strength and mobility problems are common in people experiencing homelessness [5, 6]. A single centre, cross-sectional study, which applied a broad test battery of physical functioning tests to people experiencing homelessness admitted for inpatient care, demonstrated that despite a low median age of 45 years, 83% of participants had mobility problems and 70% were frail or pre-frail [5].

Frailty, a concept normally associated with geriatric populations has been identified in younger populations across a number of settings [7] and it is recognised that those living in areas of greater deprivation experience the earlier onset of illness and associated disability [8, 9]. A high prevalence of frailty has been identified in this group [5, 10–14]. Poorer physical health and frailty means people who are homeless have less options for moving to independent housing due to accessibility issues. This reinforces the cycle of entrenched homelessness, rough sleeping, and dependence on long-term hostel accommodation [15].

Key drivers of physical frailty are poor nutritional intake and sedentary behaviour. Food insecurity is extremely prevalent among people experiencing homelessness [16] and may contribute to frailty. It is possible that protein supplementation after exercise may optimise protein synthesis rates [17] and help stabilise frailty and physical de-conditioning [18]. This has been successfully demonstrated in frail older people [19]. It has been shown that exercise, in people with substance use disorder, can increase abstinence rates and can reduce withdrawal and anxiety symptoms [20].

It is known that people experiencing homelessness, despite often having complex healthcare needs, have difficulties accessing mainstream primary healthcare services [21, 22]. Many are not registered with a GP or dentist [21] and few access physiotherapy services [22], resulting in a high rate of unscheduled and emergency care [23]. This is due to reasons such as the absence of a fixed address, difficulty keeping appointments and a lack of understanding of why the service is required and what it entails [21, 22]. People experiencing homelessness are therefore hard to reach, and healthcare systems are not designed to meet their needs. A patient-centred, flexible and low-threshold approach is advocated [24] which embodies awareness of the concerns and complex needs of people who are homeless in order to provide appropriate and timely care. Structural changes in service delivery have been proposed in the form of easy access, drop-in services [25]. However, is not clear how a flexible exercise programme with nutritional programme would work in practice for this group.

The primary aim of this study was to explore the feasibility of a low-threshold, drop-in physical rehabilitation programme with protein supplementation to target frailty and poor physical functioning in people experiencing homelessness by evaluating the recruitment and retention rates, adherence to the exercise sessions and protein supplement, evaluation of characteristics of non-returners, sporadic and frequent attenders, any adverse effects, and programme feedback. The secondary aim was to assess programme effectiveness by evaluating outcomes of grip strength, muscle mass, lower extremity physical function, pain, frailty, and risk of malnutrition.

## Materials and methods

The main recruitment site for this 16-week prospective single-arm cohort study was Merchants Quay Ireland (MQI), in the Riverbank centre, a day-service for people who are homeless and have addiction issues which is located in Dublin city centre. This setting offers services such as daily meals, medical and nursing care, needle exchange and accommodation advice. A dedicated exercise room was allocated for the intervention, which took place from February- June 2022. Following expression of interest from service users in a recently opened satellite female-only centre, an additional one-day programme was delivered in this site. Ethical approval was granted by the Faculty of Health Sciences Research Ethics Committee at Trinity College Dublin (Ethical Approval Reference Number: 211202). Data was pseudo-anonymised, with the key code kept in a separate secure location. Inclusion criteria were all clients (>18 years) accessing services in MQI who consented to participation. Any participants with acute, problematic behavioural issues or confusion, those in an agitated state or with major physical impairments (medical or orthopaedic) which precluded ability to safely participate in in the exercise class as well as those with a confirmed pregnancy were excluded from study participation.

### Procedure

Information about the study was widely distributed in MQI. Clients who were interested in finding out further information about the study were directed to ‘drop into’ the designated exercise room. All questions about the study were answered and if interested, and eligibility criteria were fulfilled, written consent was provided prior to commencement of the programme. The ‘low threshold’ features of the intervention were the rolling aspect and the ‘drop in’ nature of the programme. The rolling aspect meant that participants could join at time-point during the programme. ‘Drop’ in meant participants could ‘drop in’ at any time of the day from 10am - 4pm. In some cases, if there was no space, the client was accommodated as soon as possible. Using a psychologically and Trauma Informed approach to care [26] and based on experience from a previous Inclusion Health undergraduate clinical placement [27], the approach to participants embodied the following; empathy, building trust, open mindedness and flexibility.

The intervention was supervised and delivered by a Research Physiotherapist (FK) with 1–2 Assistants. The frequency of the programme was once-weekly over 16 weeks. The intensity of the workout was gauged visually and Borg Ratings of Perceived Exertion scale was applied [28]. Participants were advised to complete core exercises at a rate of between 11 and 13 on the Ratings of Perceived Exertion scale (RPE) scale, at a subjective level of ‘fairly light’ to ‘somewhat hard’, where they find it hard to have a conversation but can comfortably continue to exercise. This applied to both the aerobic and resistance exercises. If the participant attended regularly and was responding well to the exercise regimen, the exercise intensity was increased incrementally, so the participant then exercised at a level of 13–15 (somewhat hard to hard). The exercise level was adapted based on the results of the initial assessment, ability of participants, response to exercises and reported BORG scale as well as the clinical judgement of the Research Physiotherapist. Each exercise session commenced with a warm-up and ended with a cool-down and stretches. The exercise type consisted of eight ‘core’ resistance, aerobic and functional exercises, which were individually adapted (Table 1). The exercise programme took 20 to 30 minutes. Music was self-selected by participants. Participants were educated about physical activity recommendations and encouraged to return weekly to progress the exercise intervention. Repeat assessments were undertaken at each visit.

**Table 1:** Core intervention exercises.

Core exercise	Adaptations*
Sit to stand	Bilateral support, unilateral support, no support, use of weights, squats with UL (extensions) weights
Elbow Bends	With weights/dumbbells
Trunk mobility	(1)trunk rotations (2) sit on chair-punch air (R)® hand to (L) foot and then reach to sky to the®(R) side (diagonal movement)
	Progression-speed/precision/repetitions/weights/ from sitting or standing position
Aerobic activity	Walk/jog on spot, increase knee height/step length/speed; use of ladders, jumping jacks, mountain climbers
Side steps	Abduction-step®to (R), at same time abduct arms with theraband, back to centre, repeat to (L).
Arm raises +/or scapular strengthening	Weights/dumbbells/theraband
Step ups	Progression-low/mid/high step, bilateral, unilateral, no support/use of hand weights
Ball throws	Progression-underarm, overarm, greater force/distance thrown/single leg stance

UL; upper limb, r; right, l; left, *adaptations individualised and progressed for each participant by research physiotherapist

To promote post-exercise muscle protein synthesis, a ready-to-drink 200ml commercial nutritional supplement which consisted of 20g of protein and 400kcal was offered to all participants immediately post exercise (fresubin®, Fresenius Kabi Deutschland GmbH, Germany). The choice of a supplement rather than a “food first” approach was pragmatic and based on shelf life, the lack of preparation/equipment required, and the likelihood of acceptability to participants.

### Primary outcomes

Primary outcomes of recruitment rate, retention rate, adherence to the exercise intervention, adherence to the protein supplement, adverse events and programme feedback were measured as outlined in Table 2. Feedback was transcribed by FK and repeated back to each participant to verify accuracy.

**Table 2:** Description of primary outcome measures.

Recruitment rate	Median number of new service users who joined the programme each week
Retention rate	Rate of return visits, which was calculated from the total number of sessions which a participant could potentially attend
Adherence to the exercise intervention	Percentage of participants who completed all classes (depending on start date)
Adherence to the protein supplement	Percentage of participants who took and finished the protein supplement drink
Characteristics of non-returners, sporadic and frequent returners	Non-returners- did not return after initial evaluation,
	Sporadic attenders–attended ≤50% of available sessions,
	Frequent attenders–attended ≥50% of available sessions
Adverse events	Any accidents/injuries/or other adverse consequences of the programme
Programme feedback	Authentic feedback was sought to seek participant responses to the programme. Open text responses to the following questions were sought (1) **“What are your views on your own health, (2) ’’What are your views on any unmet physical health needs”and (3)** ’**’What are your thoughts about the programme’’,** were sought verbally and transcribed.

### Secondary outcomes

Secondary outcomes outlined in Table 3 were measured at each interaction where possible. Upper limb muscle strength and circumference girth were measured [29]. Mid-calf circumference girth was measured as it correlates with appendicular muscular mass [30]. Mid-arm muscle circumference, reflecting both muscle mass and caloric and protein adequacy, has been recommended for use in physical testing of people experiencing homelessness [31] due to the high prevalence of lower limb swelling in people with substance use disorder [32]. The Short Physical Performance Battery [33] was used to assess physical performance. Nutritional status was assessed by using the Mini-nutritional assessment (MNA) score [34, 35]. As this test has not been validated for this population, the terminology of two of the questions of the MNA (regarding acuity of illness and the presence of a neuropsychological problem) were slightly modified for the purposes of this study, i.e. “Have you recently been sick or in hospital?” and “Have you problems with concentration or memory?”. Frailty was assessed in two ways; using the Clinical Frailty Scale (CFS) [36] and the SHARE-FI [37]. Participants were also asked about the existence, location, and duration of pain. Severity of pain was assessed using the Numerical Rating Scale (0 to 10).

**Table 3:** Description of secondary outcomes, derived from [31].

Test	Construct Measured	Performance-Based Measure/Assessed by Tester	Test Description	Scoring/Unit of Measurement	Interpretation	Reference/Comparative Values
Digital Hand Dynamometer [38]	Grip strength	Performance-based measure	Performed in a sitting position while the hand was unsupported with the elbow at 90° flexion and the underarm and wrist in neutral positions. Three measurements performed with each hand. An average of highest value for right and left sides is used for analysis.	Dynamometer score (kg)	Higher scores indicate better strength	Reference average handgrip strength values [39] for men aged 30–49 are 54 kg and women are 34.5 kg, and for 65–69 years of age, average handgrip strength are 44 kg for men and 28 kg for women
Lower limb circumference measurement	Muscular mass	Assessed by tester	Girth of mid-point calf circumference measured	Width (cm)	Higher score indicates higher levels of muscular mass	The cut-off for decreased muscle mass in the elderly has been identified as 34 cm for men and 33 cm for women [30]
Upper arm circumference measurement	Muscular mass	Assessed by tester	Girth of mid-point upper arm circumference measured	Width (cm)	Higher score indicates higher levels of muscular mass	Cut-offs in the range of ≤23·5 to ≤25·0 cm could serve as a screening indicator for underweight in men and non-pregnant women [29]
Short Physical Performance Battery (SPPB) [33]	Lower extremity physical function	Performance-based measure	Consists of 3 tasks: (i) a balance task, (ii) 5 timed chair stands, (iii) a short, timed walk	0–12	Higher scores indicated better performance	<10: indicates one or more mobility limitations [33], ≤8: indicates sarcopenia [40]
Numerical Rating Scale	Pain	Self-report	Tester asks participant if they are experiencing any pain which is rated on a numerical scale	0 (no pain) to 10 (worst pain imaginable)	Higher score indicates more pain	In chronic musculoskeletal pain, <3.4 indicates mild pain, 3.5–7.4: moderate pain, ≥7.5: severe pain [41]
Clinical Frailty Scale (CFS) [36]	Frailty	Assessed by tester	Each point on the scale is correlated with a description of frailty along with a visual chart to aid the tester in classifying frailty	1 (very fit) to 9 (terminally ill)	Higher scores indicate higher levels of frailty	Not applicable
SHARE-FI Frailty Instrument [37]	Frailty	Assessed by tester	Consists of questions about exhaustion, loss of appetite, walking difficulties and low physical activity	Frailty category of non-frail, pre-frail and frail generated	Category indicates level of frailty	Not applicable
Mini-nutritional assessment [34, 35]	Risk for mal-nutrition	Assessed by tester	6 questions on food intake, weight loss, mobility, psychological stress, or acute disease, the presence of dementia or depression, and body mass index		Score ≥ 12 indicates acceptable nutritional status	Not applicable

### Statistical analyses

The difficulties of generating accurate sample size calculation for feasibility studies are acknowledged, and a minimum sample size of 24 is recommended [42], therefore the aim was to recruit a minimum of 29 participants, allowing for a 20% potential drop-out rate. Descriptive statistics were used to summarise participant demographics and feasibility measures. Nominal or ordinal variables were reported as frequencies and percentages. Continuous variables were summarised as mean and standard deviation if normally distributed and median and inter-quartile range if non-normally distributed. Data was tested for normality using the Kolmogorov–Smirnov test. Results were compared to evaluate change over time from initial to final intervention. Normally distributed data were compared from initial to final recorded time-points using paired t-tests and non-normally distributed data via the Wilcoxin-sign rank test. A complete case analysis was undertaken due to the feasibility focus of the study. Baseline results and final outcomes of participants who returned and completed >1 intervention were recorded and compared. Data was analysed using IBM SPSS V28 and p< 0.05 was considered significant. As text responses did not meet criteria for qualitative analyses in terms of richness and exploration of context [43], text responses were grouped and categorised meaningfully.

## Results

**Fig 1:**
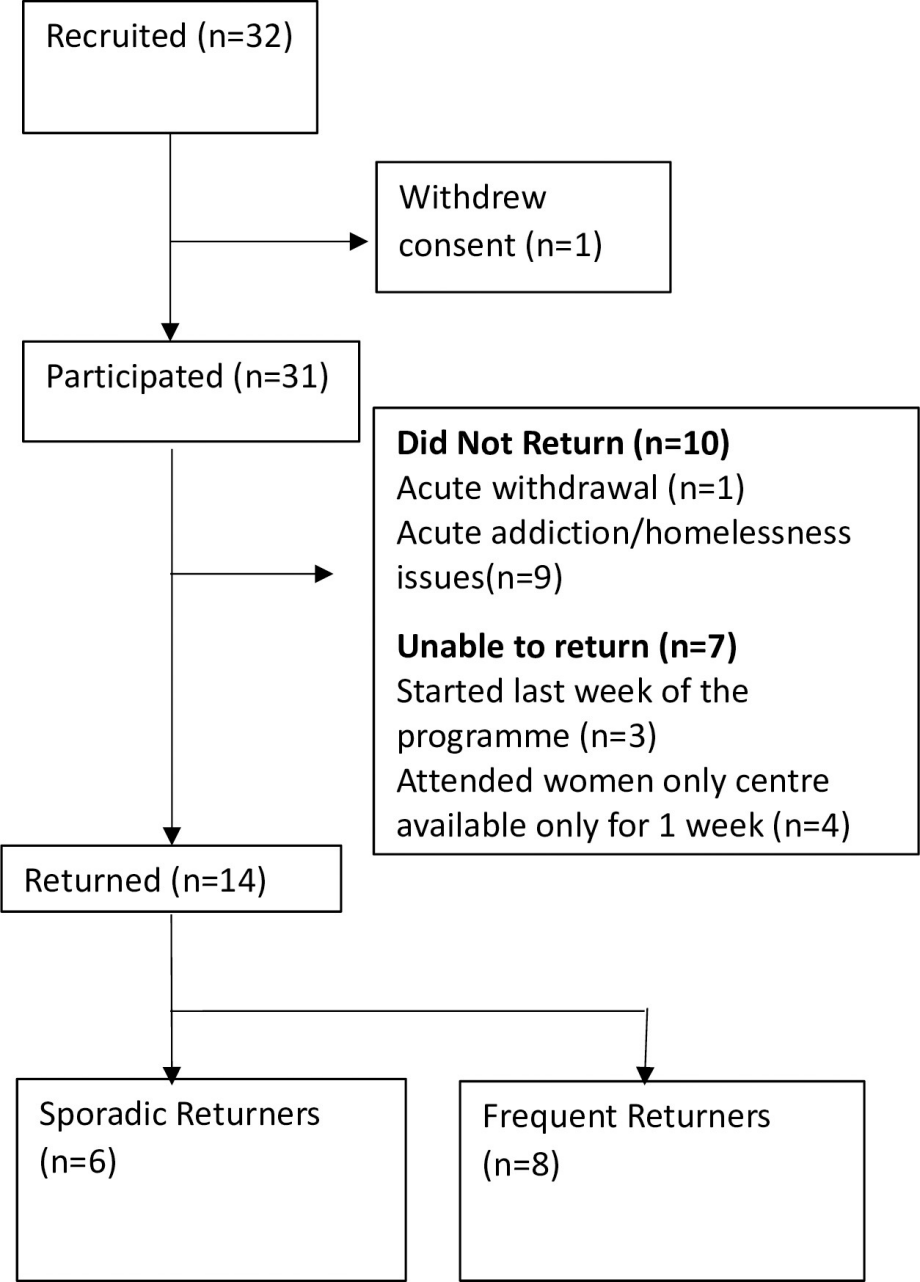
Flow diagram of participants through the study.

Thirty-two participants were recruited (Fig 1). Thirty-one participants completed the initial assessment and participated in at least one exercise session. The demographic characteristics of participants are shown in Table 4.

**Table 4:** Demographic characteristics of participants (n = 31).

Variable	N	%
**Sex**		
Male	20	64.5
Female	11	35.48
Transgender	1	3.2
**Living arrangements**		
Hostel accommodation	15	48.38
Rough sleeping	3	9.6
With family/friends	2	6.45
Hotel /B&B	2	6.45
Council apartment/house	9	29
**History of substance use**		
*Substance abuse disorder	14	45
Alcohol abuse disorder	6	19.35
No reported history of substance use	12	38.7
**Self-reported mental health issues**	19	61

*Substance abuse disorder; includes drug/alcohol abuse

### Primary outcomes

Seventy-four exercise sessions were delivered in total. Twenty-six participants, mean (SD) age 45(16) years, were recruited from the main day-service centre. Five females participated from the satellite female-only centre.

#### Recruitment rate

The median (IQR) recruitment rate was 2(1 to 3) per week, varying from week to week with a minimum of 0 to a maximum of 5 new participants recruited.

#### Retention rate

Fourteen participants returned for at least one further visit demonstrating an overall retention rate of 45%. Of participants >50 years (n = 15/31), there was a 91% (n = 10) retention rate in the main recruitment site, with at least one repeat visit to the exercise intervention. Of participants <50 years (n = 16/31), there was a 30.7% retention rate to the main recruitment site, with at least one repeat visit to the exercise intervention. Eleven participants were female, and the retention rate was 83% in comparison to a rate of 42% in the male participants (8/19). There was one transgender participant who returned once.

#### Adherence to the exercise session

Twenty-eight (90%) participants fully adhered to the initial exercise intervention, engaging in the majority of the exercise sessions. The mean (SD) time spent in active exercise was 19.4 (5.8) minutes, (minimum 10 minutes, maximum 30 minutes). Participant time commitments as well as initial assessment results influenced the delivery of a shorter session (≤ 15 minutes) in some cases (n = 6).

#### Adherence to the protein supplement

All participants (100%) consumed the Fresubin® protein supplement immediately following the exercise intervention.

#### Characteristics of participants who had the opportunity to return stratified into non-returners, sporadic returners and frequent returners

Forty three percent (6/14) were sporadic returners, returning for ≤50% of exercise classes which they could attend, which was calculated based on week started. The majority (8/14 or 57%) returned for ≥50% of exercise classes, which again was calculated based on the commencement date. Individual return rates of participants to the programme are shown in S1 Table. Due to the rolling nature of the programme, seven participants did not have the opportunity to return as they attended the one-day only intervention in the women’s centre or they attended on the last day of the programme. A remaining 10 (32%) participants, who could return, never returned after their initial assessment.

The characteristics of the sporadic returners, frequent returners and non-returners are shown in Table 5. The mean (SD) age varied between groups (p = 0.01). The mean (SD) age of frequent returners [58.4(11.9) years] was older than sporadic returners [48.7 (16) years] (p = 0.046) and those who did not return [32.1(9.6) years] (p = 0.001). Females adhered better with an 80% retention rate (5/6) where return visits were possible, (ie. excluding those who attended the one-day only service or attended on the final day) with at least one repeat visit to the exercise intervention. The corresponding figure for males was 42%(n = 8). There was one transgender participant who returned once.

**Table 5:** Demographic characteristics of participants who had an opportunity to return stratified into those who did not return, sporadic returners (attended ≤50% of available sessions) and frequent returners (attended ≥50% of available sessions).

	Did not return (n = 10)	Sporadic returners (n = 6)	Frequent returners (n = 8)
Age, years,			
Mean (SD),	32.1 (9.6)	48.7 (16)	58.4 (11.9)
range	22–53	30–71	50–77
Gender	9 males,	4 males,	4 males,
	1 female	1 female,	4 females
		1 transgender	
Living arrangements	Hostel (n = 7)	Hostel (n = 3)	Hostel (n = 4)
	Hidden homeless (n = 1)	Hidden homeless (n = 1)	Stably housed* (n = 4)
	Rough sleeping (n = 2)	Stably housed* (n = 2)	
Active addiction issues	Yes (n = 8)	Yes (n = 3)	Yes (n = 1)
	No/stable (n = 2)	No/stable (n = 3)	No/stable (n = 7)

*stably housed at the time of intervention

Those who regularly attended (n = 8) were more stable in addiction. Eighty percent (8/10) who did not return were in active addition, while the corresponding figure for sporadic returners was 50% (3/6) and for frequent returners this was 14.3% (1/7). Those who attended more frequently had the highest number of participants who were stably housed.

#### Adverse events

Three adverse events (benzodiazepine withdrawal effects, hypertension, and fatigue due to long-Covid) occurred during the assessment prior to the exercise component of the intervention. No other adverse events were recorded.

### Participant feedback

To gain an understanding of acceptability of the programme, participants were asked about their views of their own health, unmet physical health needs, and thoughts about the programme. A summary of participant feedback text responses are included in S2 Table. Overall programme feedback was overwhelmingly positive.

### Secondary outcomes

Baseline, final and change scores of participants are shown in Table 6. When compared to normative values, baseline grip strength values were below mean values for the majority (90.9%, 10/11) of female participants. Baseline grip strength was below expected values for age in (52.6%, 10/19) of the male participants. Comparing initial to the final results, there were no differences in grip strength (p = 0.671). When measuring mid-arm and calf circumference, participants were not always asked to undress if it was deemed intrusive. In these cases, measures were recorded through clothing. Five participants (16.1%) demonstrated moderately low and three (9.6%) participants severely low calf circumference measures [29]. All of these participants were male. Six (19.6%) participants were at or below the cut-off point for upper limb circumference measurement [30]. These participants were also male. Twenty-two (70.9%) participants scored 10 or above on their initial SPPB assessment, while sixteen (51.6%) participants scored the maximum score of twelve. The possible presence of sarcopenia (≤ 8 on the Short Physical Performance Battery) was noted in 3/15 (20%) of older participants (> 50 years). There were no changes to SPPB scores between baseline and final scores (p = 0.221). Baseline MNA scores demonstrated that 15 (48.4%) participants had a normal nutritional status, 15 (48.4%) were at risk of malnutrition and one (3.2%) participant was malnourished. There were no overall changes between MNA at baseline and final scores (p = 0.082). Observing baseline frailty measures, sixteen (51.6%) participants were deemed as non-frail (ie. ‘v fit’, ‘well’ and ‘managing well’ categories in CFS), eight (25.8%) pre-frail (‘vulnerable’) and seven (22.6%) frail (‘mildly frail’) using the CFS. In contrast, when utilising the SHARE-FI, 20 (64.5%) participants were deemed as non-frail, six (19.4%) pre-frail and five (16.1%) frail. The majority of the older participants (>50 years) presented as frail (6/15 or 40%) or pre-frail (4/15 or 27%), with 5/15 (33%) non-frail. The majority of the younger participants presented as non-frail (14/16 or 88%). Overall, there were no differences in frailty scores using either method (p >0.05). The majority (19/31, 61.3%) of participants reported chronic pain, while three (9.6%) reported acute pain. Overall, there were no significant differences in pain levels between the first and final assessment of participants (p = 0.498)

**Table 6:** Change scores of secondary outcomes.

	N	Baseline score	Final score	Mean/median of difference with 95% confidence interval	P value of difference
Pain					
Visual Analogue scale, median (IQR)	9*	5 (0–8)	6 (0–9.5)	0 (0–4.0)	0.498^a^
Grip Strength					
Hand dynamometry, kg, mean (SD)	13	36.32 (9.9)	35.84 (10.2)	-0.48 (-2.9–1.94)	0.671^b^
Physical Functioning					
Short physical performance battery	13	9.54 (2.22)	10.23 (1.74)	0.69 (-0.47–1.86)	0.221^b^
Nutritional Status					
Mini-Nutritional Status (NMA)	13	13 (8.5–13)	13 (11–13.5)	1 (0–3)	0.082^b^
Frailty					
SHARE-FI, median (IQR)	13	1 (0–2)	0 (0–1)	0 (0–0)	0.098^a^
Clinical Frailty Scale, median (IQR)	13	4 (2.5–5.0)	4 (2.5–5.0)	0 (0–0)	0.564^a^

*missing data for pain as some participants felt unable to score their pain, missing data for all outcomes for 1 participant as unable to measure final data due to urgency of other commitment, ^a^Wilcoxin sign-rank test, ^b^paired t-test

## Discussion

To our knowledge this was the first study to evaluate a frailty-focussed intervention in a real-world practice setting of a day services centre for people experiencing homelessness. This study included a ‘hard to reach’ vulnerable population with the majority experiencing homelessness and acute addiction issues. The main findings indicate the feasibility of this programme demonstrated by a moderate and steady recruitment rate, high retention rates among certain sub-groups and a low rate of adverse events. Physical outcomes did not change significantly, which likely needs to be evaluated with a more intensive, higher-powered study, although findings in smaller uncontrolled studies such as this still make an important contribution in this under-researched group.

Uniquely, this intervention was designed to target poor physical functioning and frailty in people experiencing homelessness. Many physical activity interventions targeted to people experiencing homelessness have been soccer focussed [44–47], which necessitates a certain level of fitness to participate. Other lower impact interventions for in people experiencing homelessness such as gardening therapy [48] or dance [49] have been shown to be beneficial but do not specifically have a physical rehabilitation focus to address variables such as strength or fitness. Many other interventions included physical activity as a component of a larger multi-modal programme [50–52], which may have diluted its effect.

This intervention was safe, and recruitment progressed at a moderate but steady rate. Uncontrollable external factors which appeared to influence recruitment were addiction issues, urgent housing issues, medical and dental needs, and inclement weather. Despite Wednesdays being selected as an optimal recruitment day due to usual high attendance rates to the centre, it coincided with welfare benefit payment day, which may have influenced footfall to the centre, due to competing priorities. This may have been exacerbated on bank holiday weeks. High recruitment and retention rates were found in female participants. Almost half of the study’s participants were over 50 years and this group yielded the highest retention rate (90%). Younger participants, as well as those in an acute phase of substance use disorder, and sleeping rough did not return following the initial interventions or attended less frequently. Generally, stability in addiction and some level of stability in housing appeared to drive better programme engagement. We observed a high level of interest and enjoyment as a direct result of exercising and an intention to return from most participants. Some quotes from participants which highlighted the positive impact of the programme were as follows; … “*it’s like a free drug*” (P8), “*it helps to fill up me week*” (P10). “*You feel you have done something with your day*” … “*it was an aim for the day*” (P19) and “*I feel safe here”* (P20).

No studies which evaluated physical activity interventions in in people experiencing homelessness [25, 44, 47–59] focussed on feasibility outcomes, so the data from this study highlighting overall high feasibility makes an important contribution.

Baseline values indicated a need for the intervention, for example, measurements of lower limb circumference highlighted low muscle mass in over 40% of the male participants. Twenty nine percent of participants scored less than ten on the SPPB, indicative of one or more mobility limitations [33]. The presence of possible sarcopenia [40] was observed in five (16.1%) participants. This low baseline ability is not surprising given the poor physical ability previously noted in previous studies which included people who are homeless, regardless of age [5, 13].

The protein supplement was very well received by participants, and taking the supplement started a conversation around taking protein through food sources where possible. It is possible that protein supplementation after exercise may optimise protein synthesis rates [17] and help stabilise frailty and physical de-conditioning [18]. This has been successfully demonstrated in frail older people [19], although we found no change in the present study. Food insecurity is extremely prevalent among people experiencing homelessness [16] and may contribute to frailty. Levels of frailty were high among participants, although we noted differences when comparing outcomes of the two frailty tools. Using the CFS, we identified 25.8% participants who were pre-frail and 22.6% who were frail. Using the SHARE-FI, six (19.4%) were identified as pre-frail and five (16.1%) frail. This is lower than levels of frailty (55%) identified in residents of a London hostel. The differences are likely due to the participants residing in a hostel for relatively high needs, a higher mean age of 55.7 (10.0) years and measurement using the Fried Frailty criteria [13]. Similarly in a US based study which recruited participants from emergency, day and transitional shelters, also with a higher mean age of 52.4 years, using the Fried criteria, 53.3% of the sample was considered frail [10]. Using the Comprehensive Frailty Assessment Instrument, frailty was identified in 60% of people utilizing a free clinic [12]. Our differences in frailty are likely due to this being a walk in, facility which provided a full spectrum of services such as meals, accommodation, and drug services.

In addition, the difficulty of comparing difference frailty instruments is challenging. The SHARE-FI assumes a more objective and/or binary scoring system, looking at five specific physical variables, namely exhaustion, weight loss, weakness (measured by grip strength), slowness and low activity levels. It offers a composite score of frailty as well as a frail, pre-frail or non-frail status. This tool is considered useful in identifying the presence of physical frailty. The CFS, however, offers a multi-dimensional insight into frailty, enabling the functional, cognitive, and psycho-social well-being of participants to be also considered. As a result, despite some participants having high physical functioning levels, a ‘vulnerable’ or ‘mildly frail’ status was judged as the appropriate frailty category when addiction was unstable or uncontrolled and psychosocial vulnerability was present. This highlights the usefulness of different frailty tools for different purposes.

This programme did not change physical outcomes. This was not surprising due to the small sample size and low frequency of the intervention which was offered once per week and the return rate of 45%. Due to the rolling design, some participants had limited opportunity to return an adequate number of times to potentially make physiological changes. Other combined exercise/educational programmes which showed significant improvements in frailty levels in older people were conducted three times per week for up to three months, many with longer term follow-up [31, 36–38]. Our study was conducted once weekly for a maximum of 16 weeks. It is also possible that some of the outcome measures used may not have been sensitive enough to detect change initial physical functioning deficits and post-intervention changes. Comparability to other studies is limited as no studies evaluated the effect of an exercise intervention using the outcomes of strength, physical functioning and frailty underlining the uniqueness of this study.

Overall, it appeared feasible to integrate a combined exercise/nutritional programme in a day services centre for people experiencing homelessness. The complex presentation of the participants, the challenges of homelessness and addiction compete strongly with an intervention of this scale to influence change. We found a tension between a low threshold design and a programme of sufficient volume to effect physiological change. While the rolling nature of that programme built in flexibility–those starting later had limited opportunity to make changes.

Limitations of this study were the lack of a control group and small sample size. A post-factum sample size calculation was conducted on the statistical package R, based on an expected adherence of 60%, a minimal acceptable adherence of 45% and a power of 80%. This indicated that a sample size of 68 would be required, suggesting this study was underpowered, so it was not surprising that physical outcomes did not change. Although this study may potentially have been underpowered, the challenges of recruiting and retaining this ‘hard to reach’ population of people who are homeless, many with acute addiction issues, in this setting of a day care/needle exchange centre, mean even small uncontrolled studies add value within this novel research terrain. Also, a minimum sample size of 24 participants, has been proposed for feasibility studies [42], so therefore our sample size of 31 exceeded this value, and the limitations of conducting a post-factum sample size calculation must be considered.

Strengths were the low threshold programme design with inbuilt flexibility in scheduling to accommodate this ‘hard to reach’ population who have traditionally been excluded from mainstream research and delivery in situ in the real world setting of a day care facility for people with acute housing and addiction needs.

Clinical implications are that day care and other outreach services for people experiencing homelessness should consider the possible inclusion of a physical rehabilitation programme into their offering, although further studies need to be conducted to elucidate optimum programming variables. Those with acute addictions challenges and those sleeping rough require their immediate needs to be looked after before they can commit to an exercise programme, although should still not be excluded from this type of programme.

Future research should evaluate the effect of a more intensive programme, offering more exercise opportunities to promote physiological changes. The low threshold features should be maintained as they worked well in practice. Targeted programmes, focussing on recruitment of older people and females should be more closely evaluated. Future studies should be fully powered and employ a randomised controlled study to extensively evaluate effectiveness, although this may be challenging to implement in practice.

## Conclusion

This study was the first of its kind to focus on exercise and nutrition to target physical functioning and frailty in people experiencing homelessness. This study showed that a targeted exercise intervention with nutritional intervention in this cohort was safe, feasible, acceptable, and positively received. Data will provide a basis on which to design and optimise rehabilitation interventions for people experiencing homelessness and will be useful to drive evidence-based policy in this field.

## Supporting information

S1 Table Individual return rate of participants to programme. (DOCX)

S2 Table Participant feedback—Text responses. (DOCX)
