# Supplementary material for: Outcomes of the LEAP feasibility trial—A low-threshold, exercise programme with protein supplementation to target frailty and poor physical functioning in people experiencing homelessness and addiction issues
Source: PLoS One. 2024 May 31;19(5):e0301926. doi: 10.1371/journal.pone.0301926 (PMC11142616; doi:10.1371/journal.pone.0301926)
Supplement: S1 Table — (DOCX) [file pone.0301926.s001.docx]

**S1 Table 1 Individual return rate of participants to programme**

| **Participant** | **Week joined** | **No of Visits** | **Total Visits** | **Rate of return** |
| --- | --- | --- | --- | --- |
| P1 | 1 | 3 | 3/16 | 18.75% |
| P2 | 1 | 11 | 11/16 | 68.75% |
| P3 | 1 | 4 | 4/16 | 25% |
| P8 | 4 | 2 | 2/13 | 15% |
| P11 | 5 | 2 | 2/12 | 16.6% |
| P13 | 6 | 2 | 2/11 | 18% |
| P15 | 8 | 6 | 6/9 | 66.6% |
| P17 | 9 | 5 | 5/8 | 62.5% |
| P18 | 9 | 2 | 2/8 | 25% |
| P19 | 10 | 6 | 6/7 | 85.7% |
| P20 | 11 | 6 | 6/6 | 100% |
| P21 | 13 | 3 | 3/6 | 50% |
| P22 | 14 | 3 | 3/3 | 100% |
| P26 | 16 | 2 | 2/2 | 100% |
