# Supplementary material for: Outcomes of the LEAP feasibility trial—A low-threshold, exercise programme with protein supplementation to target frailty and poor physical functioning in people experiencing homelessness and addiction issues
Source: PLoS One. 2024 May 31;19(5):e0301926. doi: 10.1371/journal.pone.0301926 (PMC11142616; doi:10.1371/journal.pone.0301926)
Supplement: S2 Table — (DOCX) [file pone.0301926.s002.docx]

**S2 Table 2 – Participant Feedback - Text Responses**

Programme feedback – summary of answers to the following questions (1) what are your views on your own health, (2) ''What are your views on any unmet physical health needs”, (3) ''What are your thoughts about the programme''. Each row pertains to an individual participant response.

1. **“What are your views on your own health''**

| Aware of importance of positive mindset, but living this way is very stressful, treatment by public impacts on self-esteem. What matters most: his physical disability, foot pain which affects his mobility & independence. |
| --- |
| Mental health very poor, pain severe in the past week, mood lower because of this pain; 'just fed up'….'that water looks inviting' (referring to the river looking out the window). |
| ''I'm gone weak'', ''Not able to cope on my own'', ''I’m getting awful depressed and suffering badly with my anxiety''. |
| Concerned about potential damage to liver due to drug use, worries about having a heart attack/clots, concerned re general physical health. |
| Gets regular cramps in various body parts and has been worried by same… very concerned re calf issues following 'artery burst' after injection of crack cocaine 18 month ago… loved exercise, was very competitive, feels drugs have ruined this for him. |
| Rates physical health very poorly - breathing impacted, feels like an old woman, no quality of life, 'no life', psychological health is poor, very depressed… ‘older people look so healthy and I'm so tired, kills me to get up and move it's the smokes and all (roll ups), feel double me age’… Incontinence puts her off jumping…'Depressed out of my head' ' just sick of life'. |
| 'Incarcerated in his hostel' 'feels like prison' Says it ‘would drive you to drink’… 'Hard to have a healthy mindset' there. |
| Physical health is 'good but would like to improve it'. |
| Concerned about new chest pain/back pain. Rated quality of life as 'not very good'. |
| Marriage break-up caused great mental difficulty, 'fell straight through the floor, straight into mental ward'. |
| Gained weight, breathing difficulties, can’t tie laces as tummy is in the way, wants to lose weight and improve health. Has started in a gym. |
| More aware of need to exercise and looking after her health, feels her balance is an issue. Diagnosed with chronic obstructive pulmonary disease (COPD)… ‘Sold me cooker for crack’… Very lonely in the house with her son gone… breathless at times, struggling to keep up with friends walking and talking, takes inhaler once daily in morning… worried she might be developing arthritis. |
| 'Walking is like a drug to me'.. walks everywhere. |
| Believes in exercise for physical and mental health benefits, it has a 'calming effect’… has 'battled' with bouts of depression. |
| Struggles with fatigue. She feels 'fuzzy' in her head sometimes … describes herself as having an ‘addictive personality, all or nothing' .. 'people like us who are born defective'. |
| Has post-traumatic stress disorder and wonders 'if he has brain injury', he is triggered by aggressive speaking and shouting. |
| Very concerned about his poor health and the future. ‘my health is terrible’ physical health poor, mental health ok now but very volatile, can easily change and become 'psychotic'… talked about wanting the choice and ability to be able to end his life if it got too hard. |
| Yes, concerned about physical health, feels like an old man and wants to improve his physical health and well-being; very poor quality of life …, '30 years of age and like an old man', 'I think I'll kill myself. |
| Concerned about weight, would like to lose weight. |
| Was beaten with a shovel in 2020, knee pain since then, has limited movement, and dislocated wrist, painful, clamp on left ankle from previous fall, since assault has been walking with stick for 3 years… Has Bipolar disorder and reports going from depression to manic. |
| Has been very difficult, 'I feel like I'm in prison'. |
| Just wanted to build up confidence, and stressed she wanted to lose weight as it was the cause of a lot of their problems… wants to do exercises which focus on weight loss … , 'I can’t even walk down the road, with the pains’. |
| Fractured wrist 3 weeks ago, was wearing a sling. |

1. **''What are your views on any unmet physical health needs”**

| Chronic foot pain, pain walking, tries not to think about it. |
| --- |
| Wants to start doing more exercises for strength; wants help to manage disability…Just wants health back…wants physio/exercise to manage hip pain. |
| Physio to reduce disability, determined to improve, feels that this programme will help get him to a better place. |
| Wants to manage her pain…ongoing chronic sciatica, night pain, sometimes keeps her awake. |
| Wants to get better and ‘get out of this cycle’. |
| Wants to be bigger, is concerned about recent weight loss and reduced muscle mass/bulk. Attended gym in prison and really enjoyed same, has lost weight/muscle since out of prison as has nowhere to exercise… 'just want to attend a gym and lift weights’. |
| Just commented on wanting to sort his life out. |
| Would love to get fit, used to love exercise. Anxiety from abusive relationship x 15 yrs, in new relationship now, which is also abusive … Takes inhaler and ventolin. |
| 'I just want to get fit', I have kids and want to get physically better. |
| Fitness class is exactly what he wants; can't exercise much now due to living situation (hostel). |
| Would like to attend supervised exercises, wouldn’t exercise otherwise. |
| Thinks light exercise might help him but is worried about his back too. |
| would like to be able to engage in exercise programme but very limited due to medical condition, feels very deconditioned. |
| Exercise important to him in prison exercised every day… would like to do strengthening/aerobic exercise like in the gym. |
| Concerned about weight, would like to lose weight; would love ideas on how to exercise. |
| Would help his mental health to be better and to be more physically able… would like to improve function in left arm. |
| Has blisters on his feet-very poor worn footwear. |
| Wishes to manage hip pain. |

1. '**'What are your thoughts about the programme''**

| Overall felt improvement in the last two weeks since session. Feels much improved since the last visit. Feels there is an improvement in walking the stairs particularly. Pain is more manageable…'you never regret it when you you've exercised'. |
| --- |
| ‘Always feel better after exercise’… Feeling great after the session. Enjoyed Thin Lizzy music. Found last session 'brilliant'. |
| Enjoyed session, but feels very stiff, didn’t feel like he did much but needed encouragement to realise did well. |
| Enjoyed the class, found it challenging. |
| 'Keeps me off the streets for a while’. |
| Enjoyed class, reported 'great vibe' from it, 'dream come true' (referring to getting physio/exercise). |
| Enjoyed session, feels this exercise intervention has helped, it was brilliant the last day, would like to return…felt good for the whole day after - 'it's like a free drug'. |
| 'Great, I needed that'. |
| 'I needed that, my head was a bit wrecked this morning'; (after attending counselling service in MQI); 'I'll definitely be back'. 'it helps to fill up me week'. |
| Reports getting a great buzz from exercise. Really enjoyed the session… 'love anything to do with exercise' 'love the buzz of it'; 'something to do'. Very thankful. |
| 'To be honest that was brilliant, I had back pain before but now it’s gone'. |
| ' I was very grateful to participate today, thank you'. |
| ‘Nice to come here to see a friendly face’; really found the intervention helpful, has been climbing the stairs since he started and wants to continue, feels he should be and would like to be more active; expressed thanks, -very much appreciated the session/time. |
| Was delighted when we came over to get her involved in the programme…Liked dancing to the music, enjoys the exercises and wants to come next week. |
| 'I will come back, I enjoyed it' , 'it wasn’t too hard'… Glad he did the session. Says he likes it now and that he will come back. |
| Felt muscle soreness day after the exercise, requested to get exercises written down. Found it enjoyable. Felt the exercise helps her mood. 'you feel you have done something with your day', 'at least I have done something with my head, it helps your mind to stay healthy'. |
| ‘I feel safe here’, 'people like us who are born defective', requested an exercise leaflet. got emotional finishing. |
| Felt more comfortable with exercising back, didn’t feel much pain after the exercise. |
| Was sore day after exercise but felt good… feels exercise 'opened up lungs' …'I told some others about the exercise that it was great, they were open to it a bit but the monster addiction' was bigger’ … ‘sleeping better, does not wheeze at night time like used to’. |
| 'Made my day'. |
| About the exercise- ‘this is just the right thing to get going again’ … found it good. |
| 'This is so positive because life is so s***' |
| ''Hopefully you have started me on a journey''. |
| ''I felt healthy for a minute'' … Don’t feel as breathless as I normally feel’, 'I feel I did something'. |
| Exercises helps him clear his head, would be interested in more exercise. |
| Really enjoyed the exercise, ‘makes all the difference doing it with someone’. |
